# SENTIA: a systematic online monitoring registry for children and adolescents treated with antipsychotics

**DOI:** 10.1186/2193-1801-3-187

**Published:** 2014-04-14

**Authors:** Inmaculada Palanca-Maresca, Belén Ruiz-Antorán, Gustavo Centeno-Soto, Sara Jiménez-Fernandez, Lourdes García-Murillo, Ana Siles, Sandra Villagrá, Hilario Blasco-Fontecilla, Luis Iruela-Cuadrado, Enriqueta Roman-Riechman, Cristina Avendaño-Solá, Christoph U Correll

**Affiliations:** Department of Psychiatry, Puerta de Hierro University Hospital, Madrid, Spain; Department of Clinical Pharmacology, Puerta de Hierro University Hospital, Madrid, Spain; Department of Psychiatry, Granada University, Granada, Spain; NYU Child Study Center, New York University, New York, USA; Department of Cardiology, Puerta de Hierro University Hospital, Madrid, Spain; Department of Pediatric Cardiology, La Paz University Hospital, Madrid, Spain; Department of Psychiatry, Puerta de Hierro University Hospital, CIBERSAM, Madrid, Spain; Department of Pediatrics, Puerta de Hierro University Hospital, Madrid, Spain; The Zucker Hillside Hospital, Psychiatry Research, Glen Oaks, New York, USA; Calle Manuel de Falla, 1, 28222 Majadahonda, Madrid, Spain

**Keywords:** Antipsychotics, Adverse effects, Children, Adolescents, Registry, Pharmacovigilance

## Abstract

**Introduction:**

Despite drastic increases in antipsychotic prescribing in youth, data are still limited regarding their safety in this vulnerable population, necessitating additional tools for capturing long-term, real world data.

**Methods:**

We present SENTIA (SafEty of NeurolepTics in Infancy and Adolescence; https://SENTIA.es), an online registry created in 2010 to track antipsychotic adverse effects in Spanish youth <18 years old currently taking or initiating with any antipsychotic treatment. SENTIA collects information on sociodemographic, diagnostic and treatment characteristics, past personal medical/psychiatric history, healthy lifestyle habits and treatment adherence. Additionally, efficacy and adverse effect data are recorded including the Children’s Global Assessment Scale; Clinical Global Impressions scale for Severity and Improvement, the Safety Monitoring Uniform Report Form, Simpson-Angus Scale, Abnormal Involuntary Movement Scale, vital signs, blood pressure, and EKG. Finally, fasting blood is drawn for hematology, electrolytes, renal, liver and thyroid function, glucose, insulin, lipid, prolactin and sex hormone levels. Initially, a diagnostic interview and several psychopathology scales were also included. Patients are assessed regularly and followed even beyond stopping antipsychotics.

**Results:**

Since 01/17/2011, 85 youth (11.5 ± 2.9 (range = 4-17) years old, 70.6% male) have been included at one inaugural center. After a mean duration of 17 ± 11 (range = 1-34) months, 78.8% are still actively followed. For feasibility reasons, the diagnostic interview and detailed psychopathology scales were dropped. The remaining data can be entered in <30 minutes. Several additional centers are currently being added to SENTIA.

**Conclusions:**

Implementation of a systematic online pharmacovigilance system for antipsychotic adverse effects in youth is feasible and promises to generate important information.

## Introduction

The use of antipsychotics has dramatically increased in children and adolescents in recent years. This sharp increase stands, however, in stark contrast to the relative paucity of data from controlled investigations (Olfson et al. 2012; Vitiello et al. 2009; Correll 2008; Zuddas et al. 2011). Indeed, few methodologically sound studies exist on the safety and efficacy of second-generation antipsychotics in pediatric populations (Vitiello et al. 2009; Jensen et al. 2007). Moreover, long-term evidence for the safety of antipsychotics in children and adolescents is scant (Aman et al. 2005). Importantly, most of the antipsychotics are prescribed off-label, and age-specific dose guidelines and adverse reaction profiles are often not available in these populations (Panagiotopoulos et al. 2010). The severity of some adverse effects of antipsychotics in adults, and the few available data in children and adolescents has raised concerns about the benefit/risk ratio of antipsychotics in youth (Vitiello et al. 2009; Correll 2008). Taken together, these reasons highlight the relevance of generating novel tools aimed at collecting data on antipsychotic adverse effects in pediatric populations that reflect and are generalizable to clinical practice settings and populations.

In recent years, several networks across the world have been created to routinely assess the safety of psychotropic medications in youth (Ronsley et al. 2012; Seida et al. 2012). The Child and Adolescent Psychiatry Trials Network (CAPTN) was created to collect “real life” data of pediatric patients treated with antidepressants drugs in the US (March et al. 2004; Shapiro et al. 2009). In Canada, the Canadian Alliance for Monitoring Effectiveness and Safety of Antipsychotics in Children (CAMESA) has actively collected data on the safety of antipsychotics in youth (Pringsheim et al. 2011; Ho et al. 2011). In Europe, the Therapeutic Drug Monitoring (TADS) run by the German-Austrian Swiss “Competence Network on TDM in Child and Adolescent Psychiatry” (Mehler-Wex et al. 2009) and the Pediatric Atypical Antipsychotic Monitoring Safety Study (PAMS) in the United Kingdom (Rani et al. 2009) have been developed.

**Figure 1:**
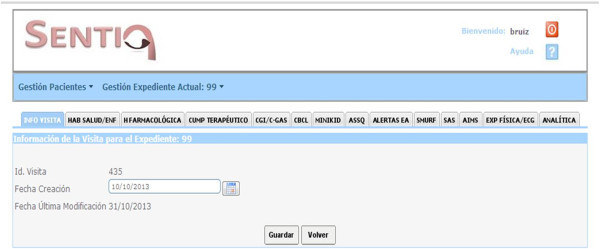
**Overall appearance of online data recording (** https://SENTIA.es **)**.

The present article is aimed at presenting SENTIA (SafEty of NeurolepTics in Infancy and Adolescence), an online registry (https://SENTIA.es) created for the long-term pharmacovigilance and safety evaluation of antipsychotics in children and adolescents in Spain (Figure 1).

### Aims of SENTIA

SENTIA was created in 2010 within the Clinical Program for Safe Antipsychotic Use in Children and Adolescents, run jointly by the Departments of Psychiatry and Clinical Pharmacology of the Puerta de Hierro University Hospital in Madrid, Spain.

The principal objective of the Clinical Program for Safe Antipsychotics in Infancy and Adolescence is to facilitate the safest use of antipsychotics in children and adolescents through the early prevention and detection of antipsychotic adverse events. Moreover, close monitoring and longterm follow-up also aims to enhance treatment adherence as well as patient and family alliance, with the ultimate goal to improve overall outcomes.

The Registry enrolled the first patient on 01/17/2011. Currently, all clinical information comes from Puerta de Hierro University Hospital (Madrid, Spain) and one referring community mental health center. After this pilot phase, SENTIA will be open to all Institutions attending children and adolescents across Spain and additional centers are currently being added.

### Description of SENTIA

SENTIA is an online database platform, which permits the registration of collected data in relation to short and long-term safety of antipsychotics in children and adolescents. This online application makes multi-centre participation possible. SENTIA is supported by grants from the Spanish Ministry of Health and Social Politics (EC10-040) and Mutua Madrileña Foundation (2010 and 2013).

SENTIA includes patients <18 years old currently taking or initiating treatment with any antipsychotic either as monotherapy or polytherapy. Patients can be recruited from different clinical settings of the participating hospitals. There are no clinical exclusion criteria. Patients with severe mental retardation or neurological abnormalities are also included, although some collected parameters are not applicable. Those who accept to be included in the online Registry are entered after the caregivers’ signing of the Informed Consent.

Children and adolescents are regularly monitored. Antipsychotic-naïve patients are monitored before starting treatment, and after 1, 3 and 6 months, and then on a 6- monthly basis. Patients included in the program who are currently on treatment for more than one month are monitored on a 6-monthly basis. The clock is reset and monitoring is carried out like in an antipsychotic naïve subject whenever a new antipsychotic is started as part of a switch or restarting procedure. Patients are followed for, before as long as they can be tracked, regardless of age, and even after the discontinuation of antipsychotic treatment and, if possible, into adulthood.

The selection of the systematically collected biological and physical safety parameters and scales was based on a literature review of possible adverse effects of antipsychotics in pediatric populations and on the recommendations of several scientific associations and expert groups dealing with children and adolescents treated with antipsychotics (Panagiotopoulos et al. 2010; Seida et al. 2012; Correll et al. 2006). We defined an adverse reaction as a response to a medicinal product which is noxious and unintended. We register all suspected adverse effects, independent from the causality category (probable, possible or unlikely) and whether it is related to the antipsychotic or to another medicinal product. If an anomaly is detected, a specialist related to the abnormal data, −e.g., cardiologist in the case of an EKG abnormality- and the patients’ physician(s), −i.e., child psychiatrist, pediatrician - are informed, so that they can take any necessary action. Thus, in addition to the regularly scheduled assessments, additional tests are performed as clinically necessary.

#### Design of SENTIA (data recording)

SENTIA comprises information on sociodemographic, diagnostic and therapeutic characteristics (age, gender, race, clinical primary diagnosis and comorbidities, target symptom(s) that motivates the antipsychotic prescription, time since these symptoms started, etc.), past personal medical and psychiatric history (i.e., drug allergies, perinatal complications, breastfeeding, psychomotor development and relevant medical history), healthy lifestyle habits (i.e., diet, physical exercise), past psychotropic use, and medication adherence, using a self-developed questionnaire to patients and to caregivers (How many doses did you miss since the last visit? Who supervises the youth’s medication taking?). Further, the following validated instruments for the assessment of the diagnosis and psychopathology of children and adolescents are included: the Mini International Neuropsychiatric Interview (Mini-KID) (Sheehan et al. 2010); Screening Questionnaire for Autistic Spectrum Disorder (ASSQ) (Ehlers et al. 1999); the Children’s Global Assessment Scale (CGAS) (Lundh et al. 2010); and the Clinical Global Impressions scale for Severity (CGI-S) and Improvement (CGI-I) (Busner and Targum 2007) and Achenbach Child Behaviour Checklist (CBCL) (Achenbach and Edelbrock 1981).

The potential adverse effects are closely monitored during each office visit using the Safety Monitoring Uniform Report Form (SMURF) (Greenhill et al. 2004), a specific adverse effect scale for pediatric psychotropic use. Given that there is not validated scale in the pediatric population, the SMURF was translated *ad hoc* into Spanish with the authors’ authorization. Furthermore, in order to better monitor any potential neurological adverse effects, we used the validated Spanish versions of the modified Simpson-Angus Scale (SAS) (Simpson and Angus 1970) and the Abnormal Involuntary Movement Scale (AIMS) (Guy 1976).

Further, the risk for metabolic syndrome, cardiovascular, cardiac, endocrine and other relevant adverse events are specifically monitored through complete physical examination, including height, weight, body mass index (BMI) and waist circumference, and sexual development, assessed using Tanner staging (Desmangles et al. 2006); an electrocardiogram (PR and QTc intervals), systolic and diastolic blood pressure and pulse rate, respectively. Finally, fasting blood work is obtained for full blood count, coagulation, serum glucose, electrolytes, hepatic and renal function tests, lipid profile, insulin, thyroid hormones, sex hormones, and prolactin.

### Ethical aspects

The Puerta de Hierro University Hospital Research Ethics Committee approved SENTIA in 2010, October. In accordance with current legislation, informed consent is obtained from parents or legal guardians and also from the patient if he/she is over 12 years old. Collected data are treated anonymously, in compliance with the local Data Protection Law (Ley Orgánica 15/1999 on confidentiality of personal data).

Personal contact details are not included in the online database. Contact details are found only on informed consent forms kept in a restricted access file. The information collected in this register is anonymous for the persons performing the data analysis or writing reports or papers since patients are identified by a six-digit code.

The project leaders have access to all included data to verify that the information entered in the database is reliable and corresponds with the data from the patient’s medical history.

The registry database is the property of the program leaders (IPM and BRA), who are responsible for any necessary organization and data analysis.

The project is registered in the European Network of Centres for Pharmacoepidemiology and Pharmacovigilance (ENCEPP). This is a new initiative established by the European Medicines Agency (EMA) which is seeking to promote the conduct of such studies and establish standards for post-marketing safety evaluations.

### Initial results

Since 01/17/2011 until 30/12/2013, 85 youth (11.5 ± 2.9 (range = 4-17) years old, 70.6% male) have been enrolled at one center. At the moment of inclusion 60% were naive to antipsychotics therapy. After a mean duration of 17 ± 11 (range = 1-34) months, 78.8% are still followed. For feasibility reasons, the time consuming diagnostic interviews (MINI-kid and ASSQ) and the CBCL were dropped. The remaining data collection is incorporated into the clinical visit and complete data entry takes <30 minutes. At present, several additional centers are being included in SENTIA.

### Limitations

A potential limitation of SENTIA is that participation is voluntary. Thus, we could potentially introduce a selection bias. We register the basic characteristics of patients prescribed antipsychotics but not entering into the registry in order to assess the representativeness of the included population. Another issue is that of missing data. Some of the required scales/measurements are clearly less time consuming and easier to comply with than others. Thus, there might be some imbalances and areas of missing data, in the sense that some information will be more easily assessed as part of SENTIA than others. As mentioned above, for feasibility reasons, we decided to drop the very time consuming interviews (MINI-kid and ASSQ) and the CBCL, focusing the clinical effort of adverse effect monitoring and global assessment of efficacy using the CGAS and CGI. The foreseen incorporation of new centers entail new challenges, such as the need for suitable procedures to guarantee quality of data: monitoring, audits as well as the need to a more mature design of the governing boards of the program, with a revised and more participative Scientific Committee. With regards to specific training to the evaluators we have chosen to skip a mandatory centralized training as we wish to apply a methodology that would be feasible and enable rolling out of SENTIA to many additional centers. Nevertheless, periodical newsletters and workshops will be scheduled as a tool to promote consistencyt.

Two major strengths of SENTIA are its standardized format and longitudinal design that will provide missing and much needed long-term safety and tolerability data, and its naturalistic design, thus giving a clear picture of real-life use and safety of antipsychotics.

## Conclusions

The field is in need of new tools that can help monitor and detect long-term, delayed and developmental adverse events of antipsychotics in pediatric patients. SENTIA, an online pharmacovigilance registry, seems to be a feasible and promising tool for the systematic monitoring of antipsychotic adverse effects in children and adolescents. In particular, the high retention rate is encouraging. The successful implementation of SENTIA in other centers needs to be tested and long-term adverse effect data will continue to be collected. Finally, routine clinical monitoring of potentially problematic long-term adverse effects of antipsychotics need to be monitored regularly in clinical practice, even outside of pharmacovigilance or research projects (Correll 2008; Pringsheim et al. 2011; Ho et al. 2011; Correll et al. 2009).

## Availability and requirements

The web address at which the database is available is https://sentia.es/gvpp/login.xhtml; use of this database is restricted to the research team. Nevertheless for any consultation or additional information to make contact with the author.
